# pH-Induced Conformational Change of the Chromophore of the Large Stokes Shift Fluorescent Protein tKeima

**DOI:** 10.3390/molecules30071623

**Published:** 2025-04-05

**Authors:** Yongbin Xu, Yun Gyo Seo, In Jung Kim, Ki Hyun Nam

**Affiliations:** 1 Department of Bioengineering, College of Life Science, Dalian Minzu University, Dalian 116600, China; 2 Key Laboratory of Biotechnology and Bioresources Utilization of Ministry of Education, College of Life Science, Dalian Minzu University, Dalian 116600, China; 3 Division of Applied Life Sciences, Institute of Agriculture and Life Science, Gyeongsang National University, Jinju 52828, Republic of Korea; 4 Department of Food Science & Technology, Institute of Agriculture and Life Science, Gyeongsang National University, Jinju 52828, Republic of Korea; 5 College of General Education, Kookmin University, Seoul 02707, Republic of Korea

**Keywords:** tKeima, chromophore, fluorescence emission, pH, conformation change, trans conformation, crystal structure

## Abstract

Fluorescent proteins (FPs) are widely used as optical probes in molecular and cell biology. tKeima is a tetrameric, large Stokes shift red fluorescent protein and the ancestral protein of mt-Keima, which is widely applied as a pH-sensitive fluorescent probe. While the pH sensitivity of mt-Keima is well characterized, the pH-dependent properties of the ancestral tKeima have not been comprehensively elucidated. To obtain a better understanding of the effects of pH on tKeima, its fluorescent emission intensity at various pH levels was measured, and its crystal structure at pH 4.0 was determined at a resolution of 2.2 Å. The fluorescence emission intensity of tKeima at pH 4.0 decreased by approximately 65% compared with its peak emission at pH 10.0. The crystal structure of tKeima at pH 4.0 revealed both cis and trans conformations of the chromophore, in contrast to previously determined structures at pH 8.0, which showed only the cis conformation. This indicates that pH induces a conformational change of the chromophore in tKeima. Both the cis and trans conformations in tKeima were stabilized by hydrogen bonds with neighboring residues. A comparison of tKeima at pH 4.0 with tKeima at basic pH, as well as with mKeima, highlights its unique structural properties. These results provide a deeper understanding of the structural basis for the pH-induced fluorescence emission changes in the Keima family.

## 1. Introduction

Fluorescent proteins (FPs) are widely used as optical probes in molecular and cell biology [1,2,3]. They are particularly valuable for their capacity to enable the tracking of target protein localization [4,5,6] or molecular interactions between biomolecules using fluorescence resonance energy transfer (FRET) [7,8,9], as well as for analyzing gene expression [10,11,12], acting as biosensors [13,14,15,16], and facilitating genome editing [17,18,19,20].

The green fluorescent protein (GFP), originally derived from jellyfish-like FPs, has a β-barrel structure containing a chromophore composed of a tripeptide [1]. Each FP has its own unique optical properties, which depend on the chromophore sequence, the residues surrounding it, FP folding, and the oligomeric state [1,21]. The fluorescence emission intensity of FPs can change depending on exogenous factors such as pH, temperature, and the presence of metal ions [22,23,24,25,26]. For example, in general, the fluorescence intensity increases at basic pH and gradually decreases as the pH deviates from the optimal level [27,28,29,30]. In addition, certain divalent metal ions induce fluorescence quenching effects, suggesting their potential application in the development of FP-based metal biosensors [22,23,31]. Among the various factors affecting FP fluorescence, pH is a crucial determinant as it influences the protonation and deprotonation states of the chromophore [1]. In GFP, which contains a chromophore composed of TYG (Thr-Tyr-Gly), the hydroxyl group of the tyrosine residue is deprotonated at basic pH but remains protonated at acidic pH [1,32,33]. These protonation-dependent fluorescence changes are significantly advantageous for pH imaging, particularly in the context of in vivo applications [34], and have been proposed as promising candidates for developing pH indicators [28]. To understand the pH-induced fluorescence changes in FPs, several crystal structures of FPs were determined under different pH conditions. Changes in pH induce configurational changes in the FP chromophore or structural changes in its surrounding environment [35]. For example, in GmKate, the quantum yields at pH 9.5 and pH 4.0 were 0.045 and 0.0023, respectively. The crystal structures of GmKate revealed that the chromophore adopted a cis conformation at pH 10 and a mixed cis/trans-conformation at pH 4.0, indicating that pH influences the configurational changes of the chromophore [34]. Meanwhile, the yellow FPs ZsYellow exhibited a fluorescent state at basic pH, but its fluorescence emission decreased to less than 40% under acidic conditions (pH < 6.0). At pH 8.0, the residues surrounding the chromophore of ZsYellow and the nearby water molecules formed a hydrogen bond network. However, at pH 3.5, in the nonfluorescent state of ZsYellow, the protonation of Glu221 disrupted this hydrogen bond network [35]. This indicates that the structural mechanisms driving pH-induced changes in fluorescence intensity vary among FPs.

The optical property of a large Stokes shift (LSS) is advantageous for fluorescence imaging applications because it reduces self-absorption due to the wide gap between the excitation and emission maxima [36,37]. Accordingly, LSS FPs are useful in multicolor imaging experiments, such as those using two-photon laser scanning microscopy, dual-color fluorescence cross-correlation spectroscopy, and FRET [37,38,39,40]. tKeima is one such LSS FP, with maximum excitation and emission wavelengths of 440 and 616 nm, respectively [37].

tKeima is the ancestor of mitochondria-targeted Keima (mt-Keima), mKeima, and dKeima. Among these, mt-Keima is a pH-sensitive, acid-stable FP used for quantifying mitophagy [41,42,43]. It is widely used in the in vivo imaging of mitophagy to understand molecular functions through pH-dependent spectroscopic changes [44,45]. The pH-induced fluorescent emission changes of mKeima and dKeima, which are derived from tKeima, have been well characterized, but the pH-induced fluorescence changes of tKeima have not been comprehensively analyzed. In addition, a previous study determined the crystal structure of tKeima at pH 8.0 and a resolution of 3.0 Å, revealing the chromophore in a cis conformation [46]; however, to the best of our knowledge, no structural information on the chromophore at other pH levels has been reported.

To obtain a better understanding of the effect of pH on fluorescence emission, the crystal structure of tKeima at pH 4.0 was determined at a resolution of 2.2 Å. At this pH, significant structural changes were observed in the tKeima chromophore, which adopted a mixture of cis and trans-conformations. This configurational change affected the environment of the chromophore, leading to alterations in its optical properties. These structural analyses not only provide detailed information on tKeima’s structure, but also deepen our understanding of the pH-induced structural changes in FPs.

## 2. Materials and Methods

### 2.1. Sample Preparation

The tKeima sample was prepared as previously reported [46]. Briefly, a DNA vector containing the tKeima gene was transformed into *Escherichia coli* BL21 (DE3) cells and cultured in LB broth supplemented with 50 μg/mL kanamycin at 37 °C in a shaking incubator. When the OD_600_ reached 0.8, protein expression was induced by adding 0.5 mM isopropyl β-d-1-thiogalactopyranoside, followed by incubation at 18 °C in a shaking incubator overnight. After harvesting the cells by centrifugation, the cell pellet was resuspended in a buffer containing 50 mM Tris-HCl (pH 8.0), 200 mM NaCl, and 20 mM imidazole and then disrupted on ice using a sonicator. The cell debris was removed by centrifugation, and the supernatant was purified using a Ni-NTA affinity column followed by size exclusion chromatography. The purified tKeima protein was stored in 10 mM Tris-HCl (pH 8.0) and 200 mM NaCl.

### 2.2. Spectroscopic Analysis

A purified tKeima solution (2.5–20 μM) was mixed with solutions of varying pH (150 μL; 0.1 M sodium citrate-HCl, pH 4.0–6.0; 0.1 M Tris-HCl, pH 7.0–9.0; and 0.1 M glycine-NaOH, pH 10–11) in a total reaction volume of 200 μL. The mixture was incubated at 25 °C for 10 min. The fluorescence intensity was visualized using an Ultrabright LED transilluminator (Maestrogen Inc., Hsinchu, Taiwan) at 470 nm. The fluorescence emissions were measured using a Synergy H1 microplate reader (BioTek, Winooski, VT, USA) with an excitation wavelength of 400 nm and an emission wavelength of 600 nm at 25 °C. All experiments were performed in triplicate.

### 2.3. Crystallization

The tKeima solution was concentrated to approximately 30 mg/mL using a Centricon (Merck Millipore, Burlington, MA, USA; cut-off: 30 kDa). The concentrated tKeima solution was stored overnight in a 4 °C incubator. tKeima crystals spontaneously grew in a 1.5-mL tube. The dimensions of the tKeima crystals ranged from 10 to 100 μm.

### 2.4. Data Collection

A 2-μL suspension of tKeima crystals was transferred from a 1.5-mL tube to a siliconized cover glass. A single tKeima crystal of approximately 100 μm in size was selected using a nylon loop and immersed in a pH exchange solution (10 μL) containing 0.1 M sodium acetate (pH 4.0) and 200 mM NaCl for 5 min. The crystal was then transferred to a fresh pH exchange solution and immersed for an additional 5 min. This procedure of immersion in the pH exchange solution was repeated four more times. Finally, the tKeima crystal was immersed in a cryoprotectant solution containing the pH exchange solution supplemented with 25% (*v*/*v*) ethylene glycol for 5 s and then mounted on a goniometer under a 100 K liquid nitrogen gas stream in the experimental hutch at the 11C beamline of Pohang Light Source II (PLS-II, Pohang, Republic of Korea) [47]. X-ray diffraction data were collected at a wavelength of 0.9794 Å at cryogenic temperatures and recorded on a Pilatus 6M detector (DECTRIS, Baden, Switzerland). The diffraction data were processed using an HKL2000 (HKL Research Inc., Charlottesville, VA, USA) [48].

### 2.5. Structure Determination

Molecular replacement was performed using the Phaser-MR program implemented in PHENIX [49] to solve the phase problem. The monomer structure of tKeima (PDB code: 8XC6), determined at pH 8.0, was used as the search model. The conformation of the tKeima chromophore was verified using an omit map. Model building was performed with COOT (version 0.9.8.95) [50]. Structure refinement and the positioning of water molecules were performed using phenix.refine in PHENIX (version 1.20.1_4487) [49] with the default parameters. The occupancy of the cis and trans conformations of the chromophores in tKeima was refined during the occupancy refinement process. The geometry of the final model was validated using MolProbity [51]. The structural figures were prepared using PyMOL (http://pymol.org; version 2.4.1; accessed on 22 November 2024).

### 2.6. Bioinformatics

The tetrameric interfaces of tKeima were analyzed with PISA [52]. Amino acid sequence alignment was performed using Clustal Omega (version 1.2.4) [53]. Structure-based sequence alignment was visualized using ESPript 3.0 [54].

## 3. Results

### 3.1. Spectroscopic Analysis of tKeima

**Figure 1:**
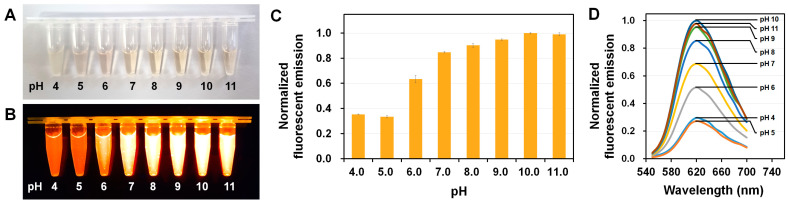
pH-induced changes in the fluorescent emission of tKeima. (**A**) Visualization of tKeima (5 μM) solutions at various pH levels. (**B**) Visualization of the fluorescence intensity of tKeima (2.5 μM) in solutions at various pH levels. (**C**) Normalized fluorescence emission intensity of tKeima in solutions at various pH levels. (**D**) The emission spectra of tKeima at various pH levels.

The optical properties of fluorescent proteins (FPs), including fluorescence intensity, are influenced by the pH environment [1]. To investigate the effect of pH on tKeima, the fluorescence emission intensity of the purified recombinant tKeima was analyzed across a pH range of 4.0 to 11.0 (Figure 1). Meanwhile, tKeima exhibited aggregation at pH 3.0, so no further analyses at this pH level were performed in this study. tKeima (5 μM) solutions appeared clear light orange under basic pH conditions but exhibited a light pink color at pH 4 and 5 (Figure 1A). When the tKeima solution was concentrated to 20 μM, it appeared clear orange at pH 8–11, whereas at pH 4 and 5, the tKeima solution exhibited an opaque pink color due to aggregation (Supplementary Materials Figure S1). When tKeima (2.5 μM) solutions at various pH levels were exposed to an LED transilluminator, tKeima’s fluorescence intensity was high at basic pH, whereas a decrease in the fluorescence intensity was observed as the pH shifted toward acidic conditions (Figure 1B). Next, the fluorescence emission intensity of tKeima at different pH levels was measured (Figure 1C). The maximum emission intensity was observed at pH 10.0. The fluorescence emission of tKeima at pH 8, 9, and 11 remained high at approximately 90.24%, 94.75%, and 99.05%, respectively, relative to that at pH 10. However, at pH 7 and 6, the relative fluorescence emission intensity decreased to 84.80% and 63.40%, respectively. Meanwhile, at pH 4 and 5, the relative fluorescence emission decreased to 35.33% and 33.39%, respectively, compared with the maximum emission intensity observed at pH 10. These spectroscopic results demonstrated that tKeima fluorescence emission is relatively high under basic pH conditions, although its intensity decreases with decreasing pH.

The emission spectrum analysis of tKeima showed that the maximum emission intensity peak was near 612 nm across all pH levels, indicating that pH affects only the fluorescence intensity, not the emission wavelength (Figure 1D).

### 3.2. Data Collection and Determination of tKeima Structure

**Figure 2:**
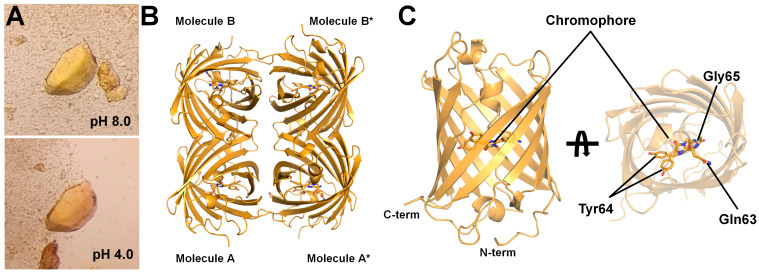
Crystal structure of tKeima^pH4.0^. (**A**) Photographs of the tKeima crystal at pH 4.0 and 8.0. (**B**) Tetrameric formation of tKeima^pH4.0^. The * indicates the neighboring TKeima molecule in crystal lattices. (**C**) Monomeric structure of tKeima^pH4.0^.

To understand the molecular mechanism underlying the pH-induced changes in fluorescence intensity, a crystallographic study was conducted to determine the crystal structure of tKeima under acidic conditions. tKeima crystals were spontaneously grown after purification and incubation at 4 °C in a buffer at pH 8.0. To prepare tKeima crystals under acidic conditions, the spontaneously grown crystals were immersed in acidic solutions with pH ranging from 3.0 to 5.0. The tKeima crystals at pH 8.0 appeared yellow under a microscope but displayed an orangeish color when exposed to acidic pH, similar to the observations in the solution state (Figure 2A). This indicates that the absorbance of tKeima in the crystal state is also influenced by pH. Since the tKeima crystal solution contained 10 mM Tris at pH 8.0, immersing the crystals in acidic solutions might not completely shift the pH to that of the acidic buffer due to the buffering capacity of Tris. To address this, the crystals were re-immersed in a fresh acidic solution with the desired pH to ensure that the local pH was at the intended level.

X-ray diffraction data were collected using tKeima crystals immersed in a pH 4.0 solution (Table 1). The tKeima crystal immersed at pH 4.0 (named tKeima^pH4.0^) diffracted up to 2.2 Å, which is a higher resolution than previously reported for tKeima at pH 8.0 (named tKeima^pH8.0^) determined at 3.0 Å resolution.

The crystal of tKeima^pH4.0^ belongs to the P2_1_2_1_2 space group, with two molecules in the asymmetric unit. These two tKeima^pH4.0^ molecules form a tetrameric structure with neighboring symmetry mates in the crystal packing (Figure 2B). The monomer structure of tKeima^pH4.0^ exhibited the typical β-barrel fold (Figure 2C), the protein folding of which is almost identical to the previously determined tKeima at pH 8.0 (see below). This indicates that the overall fold of tKeima was not affected by pH. Superimposition of the two tKeima^pH4.0^ molecules in the asymmetric unit resulted in r.m.s. deviation of 0.106 Å, indicating that the monomeric tKeima structure in its tetrameric formation is highly similar, albeit with a slight difference in the position of the chromophore (see below).

### 3.3. Chromophore of tKeima^pH4.0^

**Figure 3:**
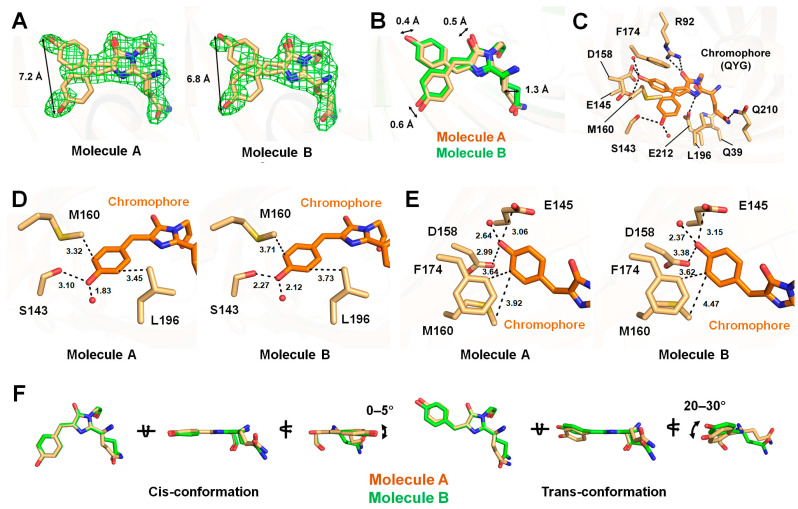
Structure of the tKeima^pH4.0^ chromophore. (**A**) The Fo-Fc omit map (green mesh, 3σ) for the tKeima^pH4.0^ chromophore. (**B**) Superimposition of two tKeima^pH4.0^ molecules from the asymmetric unit. (**C**) Interaction of the chromophore and its neighboring molecules in tKeima^pH4.0^. Close-up view of the interaction of the tyrosine ring in the (**D**) cis and (**E**) trans conformations of the tKeima^pH4.0^ chromophore with neighboring molecules. (**F**) Superimposition of the cis and trans conformations of tKeima^pH4.0^.

Analysis of the electron density map revealed that the chromophore of tKeima^pH4.0^ adopts both cis and trans conformations (Figure 3A). Occupancy refinement showed that the occupancies of the cis and trans conformations of the chromophore in tKeima molecule A were 0.52 and 0.48, respectively, with B-factors of 32.17 and 31.47 Å^2^. Meanwhile, for tKeima molecule B, the corresponding occupancies were 0.58 and 0.42, respectively, with B-factors of 30.15 and 30.06 Å^2^. The distances between the hydroxyl groups of the cis and trans conformations of the chromophore in molecules A and B were 7.20 and 6.81 Å, respectively (Figure 3A). Depending on whether the cis or transconformation of the tyrosine ring is adopted in the tKeima^pH4.0^ chromophore, the positions of the atoms in the imidazoline ring of the chromophore in molecules A and B shift by approximately 0.19–0.53 Å (Figure 3B). Although the position of the imidazoline ring of the chromophore shows a slight difference, the O_2_ and N_2_ atoms of the imidazoline ring are commonly stabilized by hydrogen bonds with the NE1/NE2 atoms of Arg92 and the OE1 atom of Glu212, respectively (Figure 3C).

In the cis conformation of both molecule A and molecule B of tKeima^pH4.0^, the hydroxyl group of the tyrosine ring of the chromophore is stabilized by hydrogen bonding with the OG atom of Ser143 and a water molecule, at distances of 3.10/2.27 Å (molecule A/B) and 1.83/2.12 Å, respectively (Figure 3D). The CE1 and CE2 atoms of the tyrosine ring are stabilized by hydrophobic interactions with the CD2 atom of Leu196 and the CE atom of Met160 at distances of 3.45/3.73 Å and 3.32/3.71 Å, respectively (Figure 3D).

In the trans conformation of both molecule A and molecule B of the tKeima chromophore, the hydroxyl group of the tyrosine ring of the chromophore is stabilized by hydrogen bonds with the OE2 atom of Glu145, the OD2 atom of Asp158, and water molecules, at distances of 3.06/3.15 (molecules A/B), 2.99/3.38, and 2.64/2.37 Å, respectively (Figure 3E). Additionally, the CE1 atom of the tyrosine ring is stabilized by hydrophobic interactions with the CE atom of Met160 and the CD2 atom of Phe174 at distances of 3.94/3.62 and 3.64/4.47 Å, respectively (Figure 3E). Despite the same interacting amino acids and water molecules in the trans conformation, the interaction distances between tKeima molecules A and B differ. This is likely due to the positional differences between the two molecules, which are influenced by the cis/trans-conformation ratio.

The conformation of the Gln side chain in the chromophore is identical in tKeima^pH4.0^ molecule A and molecule B, regardless of the chromophore conformation, except for the conformational difference observed in the C8–C10 atoms between them (Figure 3F). The OE1 atom of Gln in the chromophore is stabilized by a hydrogen bond with the NE2 atom of Gln210 at distances of 2.91/2.90–2.98 Å (molecule A/B). The NE1 atom of Gln in the chromophore is stabilized by hydrogen bonds with the OH atom of Tyr11 and the OE1 atom of Gln39, respectively, at distances of 3.30–3.42/3.48–3.53 Å and 3.40–3.51/3.66 Å.

In the side view of the imidazoline ring of the chromophore, the tyrosine ring group in the trans conformation of the tKeima^pH4.0^ chromophore is almost parallel to the imidazoline ring (Figure 3F). In contrast, in the cis conformation of the tKeima^pH4.0^ chromophore, the tyrosine ring group is rotated approximately 30° counterclockwise relative to the imidazoline ring (Figure 3F).

### 3.4. Comparison Between tKeima^pH4.0^ and tKeima^pH8.0^

To understand the pH-induced fluorescence and structural changes of tKeima, the crystal structures of tKeima^pH4.0^ were compared with the previously determined structure of tKeima^pH8.0^ (PDB code: 8XC6) [46]. The superimposition of the monomeric and tetrameric forms of tKeima^pH4.0^ and tKeima^pH8.0^ showed r.m.s. deviations of 0.219–0.231 Å and 0.517 Å, respectively. This indicates that pH did not significantly affect the monomer structure of tKeima, but the tetrameric assembly was slightly changed. When the chain A molecules of tetrameric tKeima^pH4.0^ and tKeima^pH8.0^ were superimposed, subtle movement of the other chain molecules was observed (Supplementary Materials Figure S2), indicating that pH affects the tetramer interface of tKeima.

To investigate how pH affects the tetrameric interface of tKeima, the interactions between tKeima^pH4.0^ molecules in the tetrameric formation were analyzed (Table S1). The interaction between chain A and chain B in the tetrameric formation of tKeima is stabilized by 12 hydrogen bonds (Gly21-Glu91, Ser122-Thr103, Glu91-Gly21, Glu91-Asn125, Thr103-Thr103, Thr103-Ser122, Thr103-Ser122, Ser122-Thr103, Asn125-Glu91, Asn125-Thr177, and Thr177-Asn125). Meanwhile, the interaction between A-A* molecules in the tetrameric formation is stabilized by 16 hydrogen bonds (Glu97-Arg150, Pro142-Tyr191, Arg146-Met160, Arg150-Glu97, Arg150-His169, Asp158-Tyr159, Tyr159-Asp158, Tyr159-Glu173, Tyr159-Tyr189, Met160-Arg146, His169-Arg150, Glu173-Tyr159, Tyr189-Tyr159, and Tyr191-Pro142) and eight salt bridges (Glu97-Arg150 and Arg150-Glu97). The interacting residues of tKeima at pH 4.0 in the tetrameric form were not identical to those at pH 8.0, indicating that the tetrameric formation of tKeima slightly changed within the pH range of 4.0–8.0. The distances between the interacting residues at the tetrameric interface differed between tKeima^pH4.0^ and tKeima^pH8.0^ (Table S1). These results indicate that pH may influence the tightness of the tetrameric formation of tKeima.

The crystal structure of the chromophore and its environment were compared between tKeima^pH4.0^ and tKeima^pH8.0^. In the previously determined tKeima^pH8.0^, a partial Fo-Fc electron density map in the trans conformation region was observed, but it was unsuitable for model building due to insufficient Fo-Fc electron density [46]. This suggested the almost exclusive existence of the cis conformation of the tKeima^pH8.0^ chromophore, whereas tKeima^pH4.0^ showed both cis and trans conformations. These results indicate that pH induces a configurational change in the tKeima chromophore.

**Figure 4:**
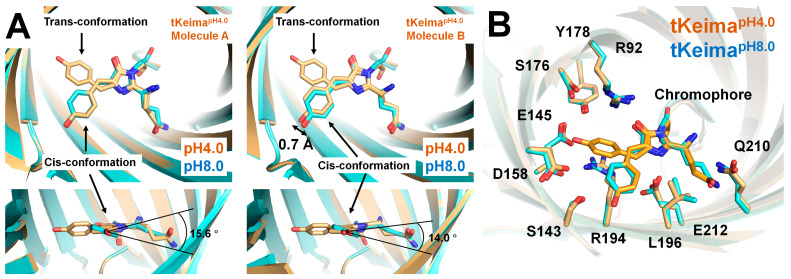
Structural comparison of tKeima^pH4.0^ and tKeima^pH8.0^. (**A**) Superimposition of tKeima^pH4.0^ and tKeima^pH8.0^ (PDB code: 8XC6) reveals differences in the positions of their chromophores. (**B**) Close-up view of the chromophore and its neighboring residues for tKeima^pH4.0^ (orange) and tKeima^pH8.0^ (cyan).

Superimposition of the monomeric tKeima^pH4.0^ and tKeima^pH8.0^ showed that the positions of their cis conformations were slightly different (Figure 4A). In the top view, the cis conformation of the tKeima^pH4.0^ chromophore from molecule A was well aligned with that of the tKeima^pH8.0^ chromophore. However, in the side view, the tyrosine ring was rotated by 15.6° between the tKeima^pH4.0^ chromophore from molecule A and the tKeima^pH8.0^ chromophore (Figure 4A). Meanwhile, in the top view, the cis conformation of the tKeima^pH4.0^ chromophore from molecule B was shifted when compared to the cis conformation of the tKeima^pH8.0^ chromophore. Additionally, in the side view, the tyrosine ring was rotated by 14.0° between the tKeima^pH4.0^ chromophore from molecule B and the tKeima^pH8.0^ chromophore (Figure 4A). Based on the electron density map, the cis conformation of the chromophore of tKeima^pH4.0^ was flexible, with low occupancy, whereas that of tKeima^pH8.0^ was rigid. This indicates that pH affects both the conformation and the flexibility of the chromophore.

In addition, subtle conformational differences were observed in most neighboring residues (Arg92, Ser143, Glu145, Asp158, Ser176, Tyr178 Arg194, Leu196, Qln210, and Glu212) in the vicinity of the chromophore and the chromophore-interacting residues (Figure 4B). These results indicate that pH affects not only the conformation of the chromophore but also its local environment. These structural changes may have occurred not only due to the effect of pH but also as a result of the averaged electron density map for the cis and trans conformations of the chromophores in tKeima^pH4.0^.

### 3.5. Comparison with Other Keima Families

**Figure 5:**
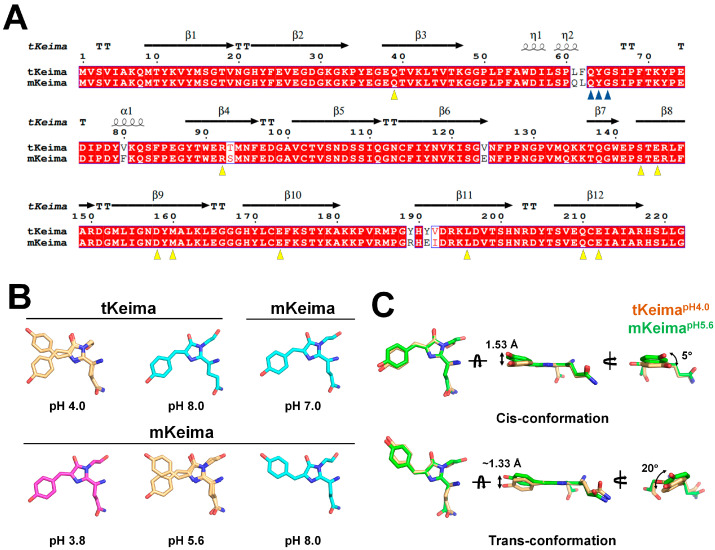
Structural comparison of tKeima and mKeima. (**A**) Sequence alignment of tKeima (UniProt code: Q1JV72) and mKeima (Q1JV70). The residues comprising the chromophore and its environment are indicated by blue and yellow triangles, respectively. (**B**) Structural comparison of the chromophore conformation of tKeima^pH4.0^, tKeima^pH8.0^ (PDB code: 8XC6), mKeima^pH3.8^ (2WHS), mKeima^pH5.6^ (2WHT), mKeima^pH8.0^ (2WHU), and mKeima^pH7.0^ (3IR8). (**C**) Superimposition of the dual cis and trans conformation chromophores of tKeima^pH4.0^ and mKeima^pH5.6^.

To obtain a better understanding of the pH-induced structural changes of the Keima family, the pH-induced configurational changes of the chromophore in tKeima and mKeima were compared. mKeima is a monomeric variant derived from tKeima, engineered through the substitution of seven amino acids (Leu61Gln, Phe62Leu, Val80Phe, Thr93Ser, Thr124Glu, Tyr189Arg, and Tyr191Glu) [37]. These substituted residues do not directly interact with the chromophore [46]. Sequence alignment and structural analysis revealed that the residues interacting with the chromophore in tKeima are identical to those in mKeima, indicating that the chromophore and its local environment remain identical between tKeima and mKeima (Figure 5A).

To date, two research groups have reported the crystal structures of mKeima in the Protein Data Bank [55,56]. Among them, Violot et al. determined the crystal structure of mKeima at pH 3.8, 5.6, and 8.0 [55], exhibiting the cis, cis/trans, and trans conformations of the chromophore, respectively (Figure 5B). Their findings demonstrated that as the pH shifts from basic to acidic, the chromophore transitions from a trans to cis conformation, with a mixed cis/trans-conformation observed at mildly acidic pH. This pH-induced configurational change of the chromophore in mKeima is similar to that in tKeima; however, the previously determined tKeima^pH8.0^ exhibits a cis conformation of the chromophore, which differs significantly from the trans-conformation observed in mKeima at the same pH [55] (Figure 5B). Meanwhile, Henderson et al. determined the crystal structure of mKeima at pH 7.0, showing a cis conformation of the chromophore [56], with a trend similar to that of tKeima^pH8.0^ (Figure 5B). Further investigations are required to clarify whether these configurational changes are dynamic or influenced by crystallization conditions.

Meanwhile, although the pH environments differ, the dual cis/trans conformation of mKeima^pH5.6^ exhibits structural properties similar to those of tKeima^pH4.0^, as determined in this study (Figure 5B). The interactions of both cis and trans conformations of the mKeima^pH5.6^ chromophore with neighboring residues were identical to those of tKeima^pH4.0^, but the interaction distances between the chromophore and its interacting residues differed.

Superimposition of the dual-conformation structures of tKeima and mKeima revealed an r.m.s. deviation of 0.259–0.307 Å. While the cis and trans conformations of the chromophore in the two proteins were similar, slight positional differences were observed (Figure 5C). In addition, superimposing the four mKeima molecules from the asymmetric unit revealed an r.m.s. deviation of 0.136–0.159 Å, indicating small positional variations among the chromophores (Supplementary Materials Figure S3). These results suggest that the positional shifts of the chromophore within the β-barrel are intrinsic properties of the chromophore itself, rather than differences between tKeima and mKeima. This finding resembles the previously reported flexibility of the chromophore of mDsRed [57].

The cis conformation of the tKeima^pH4.0^ chromophore is nearly planar, whereas the trans conformation is rotated clockwise by 30° (Figure 3C). Meanwhile, the mKeima^pH5.6^ chromophore adopts a cis conformation that is nonplanar, with a 2 Å shift in the N-terminal direction (relative to the imidazoline ring side of the chromophore) and a 5° clockwise rotation, when compared with the cis-conformation chromophore of tKeima^pH4.0^ (Figure 5C). The distance between the hydroxyl groups of the cis-conformation chromophores in tKeima^pH4.0^ and mKeima^pH5.6^ molecules was ~1.53 Å. The trans-conformation chromophore of mKeima^pH5.6^ is rotated counterclockwise by 20° compared with that of tKeima^pH4.0^ (Figure 5C). The distance between the hydroxyl groups of the trans-conformation chromophores in tKeima^pH4.0^ and mKeima^pH5.6^ molecules was ~1.33 Å. These results indicate that the position and rotation angle of the tyrosine ring group in both the cis and trans conformations of the chromophore differ significantly between tKeima^pH4.0^ and mKeima^pH5.6^.

Direct structural comparison between tKeima and mKeima is not appropriate because their amino acid sequences are not identical, and the pH conditions applied during data collection differ. Nevertheless, comparison of the dual conformations of the chromophore in tKeima and mKeima suggests that the position and conformation of the chromophore’s tyrosine ring region within the β-barrel structure can vary.

## 4. Discussion

pH affects the fluorescence emission intensity of FPs, and their optical properties can be utilized as FP-based pH indicators. Among the various FPs, tKeima-based mt-Keima is widely applied in in vivo imaging studies. To obtain a better understanding of the molecular properties of the pH-induced fluorescence change, spectroscopic and structural analyses of tKeima were performed. The spectroscopic analysis revealed that the fluorescence intensity was relatively high at basic pH, while it was reduced by 65% at pH 4.0. Despite this reduction in intensity, the fluorescence emission of tKeima did not completely disappear. This suggested that both fluorescent and nonfluorescent structural states of tKeima may exist at acidic pH. The crystal structure of tKeima^pH4.0^ exhibited both cis and trans conformations, which is distinct from the previously reported cis conformation of the tKeima^pH8.0^ chromophore. This indicates that pH induces a conformational change in the chromophore of tKeima. Based on the spectroscopic and structural analyses, the cis and trans conformations of tKeima represent the fluorescent and nonfluorescent forms of the tKeima chromophore, respectively.

Previous observations of the photoactivatable FPs suggested that the cis and trans conformations of the FP chromophore correlated with the neutral and anionic states of the chromophore [58,59,60]. Based on the crystal structures of tKeima^pH4.0^ and tKeima^pH8.0^, the following molecular mechanism for the configurational change of the tKeima chromophore induced by the pH change can be proposed. At a basic pH of 8.0, the anionic chromophore of tKeima forms a hydrogen bond with the hydroxyl group of Ser143. This Ser143 residue also forms a hydrogen bond with Asp158, which does not interact with the tKeima chromophore. Although the complete trans conformation of tKeima has not been observed, the crystal structure of tKeima at pH 4.0 suggests that the protonation environment induces a change in the chromophore from a cis to a trans conformation. Considering the pKa values of tKeima (6.5) and Asp158 (3.8), the phenolic chromophore of tKeima would be expected to form a hydrogen bond with deprotonated Asp158, stabilizing the trans conformation of the chromophore at pH 4.0.

Consequently, changes in the protonation/deprotonation state of the chromophore due to pH are likely to induce configurational changes in the chromophore to stabilize the hydrogen bonding. As mentioned earlier, the chromophore and surrounding amino acids of tKeima and mKeima are identical. This interpretation of the cis-conformation structure of tKeima at basic pH aligns with the findings of Henderson et al., who reported the cis conformation of the mKeima chromophore at pH 7.0 [56].

However, unlike tKeima, which adopts a cis conformation at basic pH, Violot et al. reported that mKeima adopts a trans conformation under the same conditions [55]. They suggested that, at pH 8.0, deprotonated Asp158 forms a hydrogen bond with the hydroxyl group of Ser143, which prevents these residues from stabilizing the anionic chromophore. Instead, the negatively charged side chain of Asp158 promotes the binding of the phenolic form of the chromophore in its trans conformation. At pH 3.8, the protonated Asp158 forms a hydrogen bond with Ser142, allowing the hydroxyl group of Ser143 to bind the anionic chromophore in its cis conformation [55]. At pH 5.6, the chromophore configuration of mKeima appears as a mixture of cis and trans forms.

In conclusion, although it is structurally evident that the chromophores of tKeima and mKeima undergo pH-dependent configurational changes, the underlying reason for their differing conformations at the same pH remains unclear. To obtain a better understanding of these pH-dependent structural changes, it will be critical to determine the crystal structure of tKeima at pH < 3.8 under conditions where Asp158 is protonated. Additionally, further studies are required to investigate whether amino acids other than Ser143 and Asp158 influence the structural changes of the chromophore.

In the asymmetric unit, the occupancy levels of the cis and trans conformations of the tKeima chromophore in chain A were 0.52 and 0.48, respectively, while in chain B they were 0.58 and 0.42. This indicates that the conformation of the tKeima chromophore does not follow a uniform distribution at the same pH and that changes between the cis and trans conformations can occur. In addition, the electron density map of the tyrosine ring of the chromophore observed in this study was not sufficiently clear to define the positions of each atom. Therefore, the distances at which the tyrosine ring of the chromophore and the surrounding amino acids and water molecules interact may not be accurate. Furthermore, although the cis and trans conformations of the tKeima chromophore interact with the surrounding amino acids and water molecules in the electron density maps, these maps represent the average electron density from the cis and trans conformations of the tKeima chromophore and the surrounding molecules. Accordingly, rather than focusing on the absolute distances of interaction between the chromophore and neighboring molecules, greater emphasis should be placed on the structural changes of the chromophore and its surroundings induced by configurational changes.

In this study, our results demonstrated the pH-induced isomerization of tKeima between pH 4.0 and pH 8.0. To provide more precise structural insights, high-resolution structures of tKeima at both acidic and basic pH, along with intermediate pH levels, will be required. In the tKeima chromophore, the double bond formation between Tyr Cα and Cβ restricts free rotation, requiring a large amount of energy for isomerization. Accordingly, to accurately determine the structural mechanism underlying the pH-induced cis-to-trans configuration change of the chromophore, further studies, such as time-resolved chemical mixing experiments, may be required.

In conclusion, based on our spectroscopic and structural analyses, our results suggest that the cis conformation of the tKeima chromophore at basic pH is associated with the fluorescent state. When tKeima shifts to an acidic pH, the hydrogen bond network of the cis conformation is disrupted, inducing a structural rearrangement of the chromophore into the trans conformation. This transition leads to a nonfluorescent state of the tKeima chromophore, thereby reducing fluorescence intensity.

## Figures and Tables

**Table 1:** Data collection and structure refinement statistics.

Data Collection	tKeima^pH4.0^
Synchrotron	Beamline 11, PLS-II
Wavelength (Å)	0.9794
Space group	P2_1_2_1_2
Unit cell	
a, b, c (Å)	70.005, 85.773, 109.759
α, β, γ (°)	90.0, 90.0, 90.0
Resolution (Å)	50.0–2.20 (2.24–2.20)
Unique reflections	34,083 (1658)
Completeness (%)	99.0 (98.9)
Redundancy	7.9 (6.3)
Mean *I*/σ(*I*)	14.0 (2.58)
R_merge_	0.131 (0.486)
CC1/2	0.998 (0.844)
CC*	0.997 (0.957)
**Refinement**	
Resolution range (Å)	46.23–2.20 (2.26–2.20)
*R*_work_	0.2312 (0.3493)
*R*_free_	0.2747 (0.3698)
R.m.s. deviations	
Bonds (Å)	0.007
Angles (°)	1.104
Average *B* factors (Å^2^)	
Protein	27.42
Water	30.89
Ramachandran plot (%)	
Most favored	98.85
Allowed	1.15
Outlier	0
PDB code	9LPU

Values in parentheses are for outer shells.

## Data Availability

Coordinates and structure-factors of the tKeima structure have been deposited in the PDB under the accession codes 9LPU.

## Supplementary Materials

The following supporting information can be downloaded at: https://www.mdpi.com/article/10.3390/molecules30071623/s1, Figure S1: Visualization of tKeima solutions at various pH levels; Figure S2: Superimposition of tetrameric tKeima^pH4.0^ and tKeima^pH8.0^; Figure S3: Superimposition of the four mKeima^pH5.6^ molecules; Table S1: Tetramer interface of tKiema^pH4.0^ and tKeima^pH8.0^.
