# Supplementary material for: pH-Induced Conformational Change of the Chromophore of the Large Stokes Shift Fluorescent Protein tKeima
Source: Molecules. 2025 Apr 5;30(7):1623. doi: 10.3390/molecules30071623 (PMC11990504; doi:10.3390/molecules30071623)
Supplement: Supplementary file 1 [file molecules-30-01623-s001.zip › molecules-3479464-supplementary.pdf]

Supplementary Data

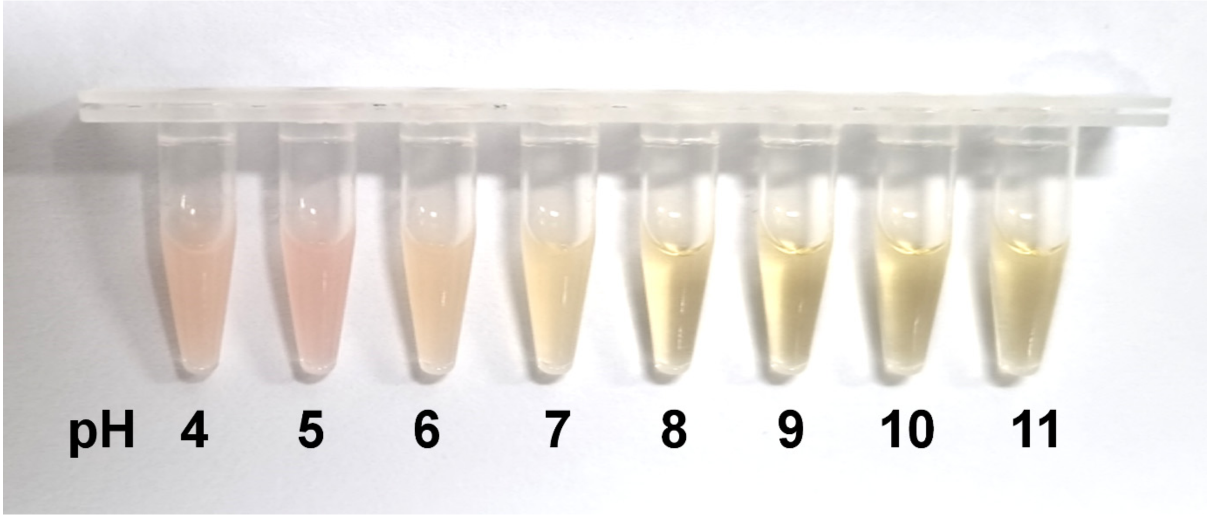

Supplementary Figure S1. Visualization of tKeima (20 μM) solutions at various pH levels.

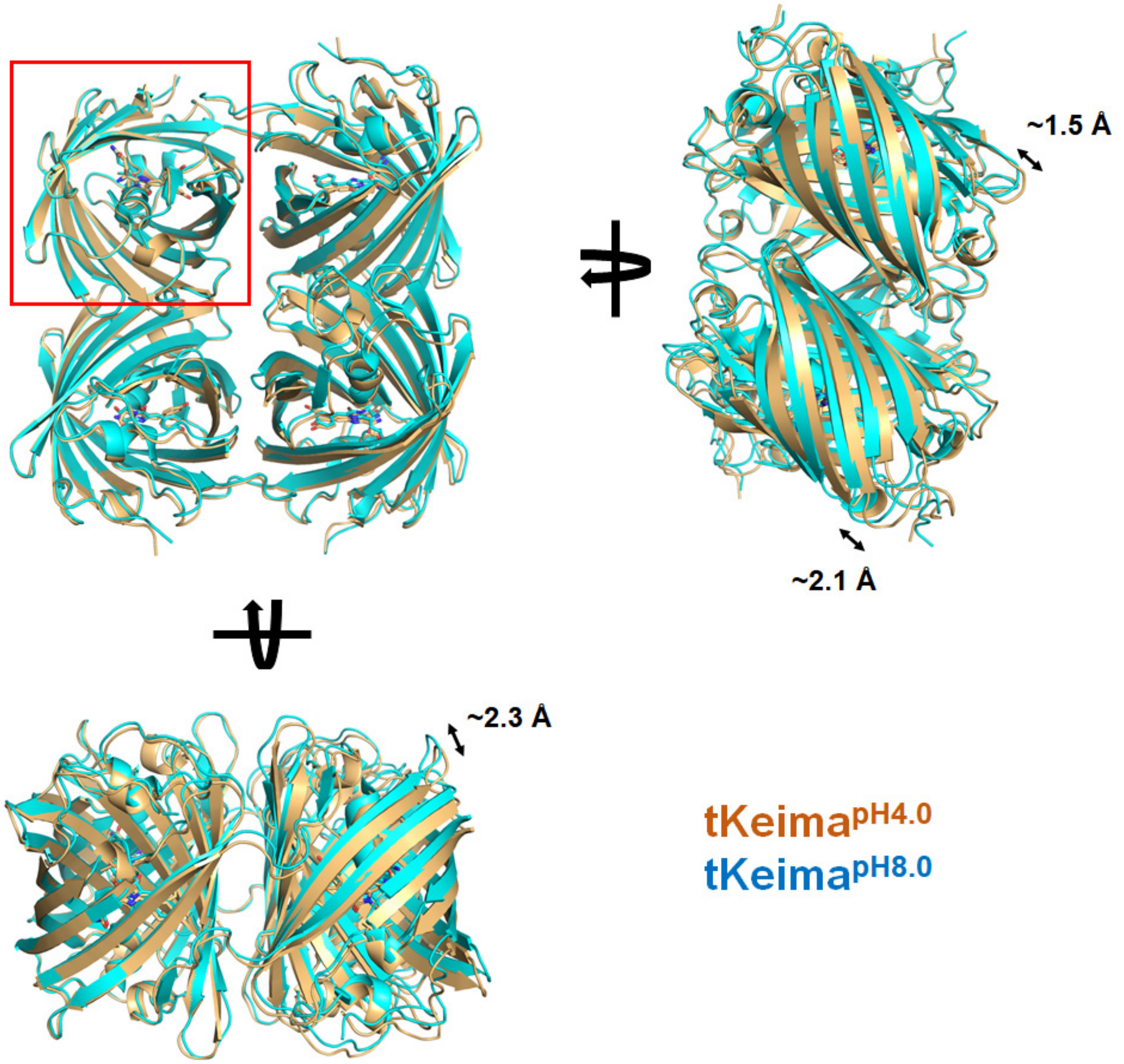

Supplementary Figure S2. Superimposition of molecule A from tetrameric tKeima$^{pH4.0}$ and tKeima$^{pH8.0}$. The aligned molecule is highlighted with a red box.

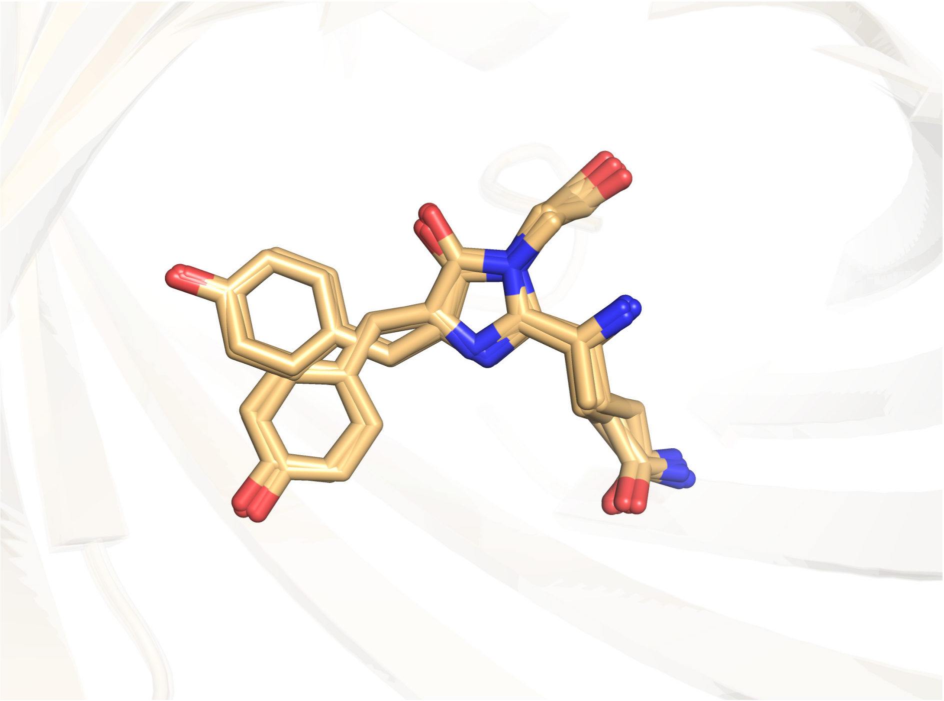

Supplementary Figure S3. Superimposition of the four mKeima$^{pH5.6}$ molecules (PDB code: 2WHT) in the asymmetric unit.

Table S1. Tetramer interface of tKiema$^{pH4.0}$ and tKeima$^{pH8.0}$.

Interface interaction	Molecule A [atom]	Molecule B [atom]	Distance (Å) tKeima$^{pH4.0}$	Distance (Å) tKeima$^{pH8.0}$
A–B Hydrogen bond	Gly21 [N]	Glu91 [OE1]	3.43	
	Glu91 [OE1]	Gly21 [N]	3.54	
	Glu91 [OE1]	Asn125 [N]	2.65	2.91
	Thr103 [OG1]	Thr103 [OG1]	2.96	2.57
	Thr103 [OG1]	Ser122 [O]	3.63	
	Thr103 [OG1]	Ser122 [OG]	2.51	2.65
	Ser122 [O]	Thr103 [OG1]	3.82	3.66
	Ser122 [OG]	Thr103 [OG1]	2.67	2.77
	Asn125 [N]	Glu91 [OE1]	2.77	2.86
	Asn125 [OD1]	Thr177 [OG1]		3.50
	Asn125 [ND2]	Glu91 [OE1]	3.63	
	Asn125 [ND2]	Thr93 [OG1]		3.31
	Asn125 [ND2]	Thr177 [OG1]	3.39	
	Thr177 [OG1]	Asn125 [OD1]	3.49	3.09
	Molecule A [atom]	Molecule B* [atom]	Distance (Å)	Distance (Å)
A–B* interface Hydrogen bonds	Glu97 [OE1]	Arg150 [NH1]	2.84	2.98
	Glu97[OE2]	Arg150 [NH2]	2.98	2.72
	Pro142 [O]	Tyr191 [OH]	2.63	2.53
	Thr144 [O]	Arg146 [NH2]		3.06
	Arg146 [NH1]	Tyr159 [O]	3.39	
	Arg146 [NH1]	Met160 [O]	3.58	2.78
	Arg150 [NH2]	His169 [O]	2.93	2.78
	Arg150 [NH1]	Glu97 [OE1]	2.84	3.15
	Arg150 [NH2]	Glu97 [OE2]	2.98	2.95
	Asp158 [OD1]	Arg146 [NH2]		3.46
	Asp158 [OD1]	Arg146 [NH1]		3.18
	Tyr159 [O]	Arg146 [NH1]	3.39	2.96
	Tyr159 [O]	Arg146 [NH1]	3.87	
	Tyr159 [OH]	Glu173 [OE2]	2.95	
	Met160 [O]	Arg146 [NH1]	3.58	
	His169 [O]	Arg150 [NH2]	2.93	3.05
	Glu173 [OE2]	Tyr159 [OH]	2.95	
	Tyr189 [OH]	Tyr159 [O]	3.87	3.82
	Tyr191 [OH]	Pro142 [O]	2.63	2.33
A–B* interface Salt Bridges	Glu97 [OE1]	Arg150 [NH2]	2.84	3.29
	Glu97 [OE1]	Arg150 [NH1]	3.55	2.98
	Glu97 [OE2]	Arg150 [NH1]	3.80	2.72
	Glu97 [OE2]	Arg150 [NH2]	2.98	
	Arg150 [NH1]	Glu97 [OE1]	2.84	3.15
	Arg150 [NH1]	Glu97 [OE2]	3.80	2.95
	Arg150 [NH2]	Glu97 [OE1]	3.55	
	Arg150 [NH2]	Glu97 [OE2]	2.98	
	Asp158 [OD1]	Arg146 [NH2]		3.46
	Asp158 [OD1]	Arg146 [NH1]		3.18
